# AGING ATTITUDES: THE EFFECTS OF INTERGENERATIONAL RELATIONSHIPS AND VILLAGE ECONOMIC CONTEXT IN RURAL CHINA

**DOI:** 10.1093/geroni/igae098.3265

**Published:** 2024-12-31

**Authors:** Xiaoyu Fu, Ying Xu, Merril Silverstein

**Affiliations:** Syracuse University, Syracuse, New York, United States; Syracuse University, Syracuse, New York, United States; Syracuse University, Syracuse, New York, United States

## Abstract

Understanding the dynamics between intergenerational relationships and attitudes toward aging is crucial for developing supportive policies for older adults, particularly in rapidly changing rural contexts (Wu & Yuan, 2023). This study investigated how parent-adult child relationships (level 1 predictors) and village economic development (level 2 predictor) influence older adults’ attitudes toward aging in rural China. From the 2021 wave of the Longitudinal Study of Older Adults in Anhui Province, we sampled 1394 older adults aged 60 and over across 64 villages in Anhui. Using mixed-effects multilevel regression models, we found that the quality of parent-adult child relationships, support from adult children, and the level of economic development in villages positively affect older adults’ attitudes toward aging. Importantly, the effect of parent-adult child relationship on aging attitude is shaped by the village’s economic development level, indicating a stronger positive association in more economically developed contexts. These findings highlight the significant contextual effect of economic conditions on the impact of intergenerational relationships on aging attitudes, underscoring the importance of considering both individual and village-level factors in aging research in rural China.

